# Mathematical modeling supports substantial mouse neural progenitor cell death

**DOI:** 10.1186/1749-8104-4-28

**Published:** 2009-07-14

**Authors:** Michael J McConnell, Hugh R MacMillan, Jerold Chun

**Affiliations:** 1 Crick-Jacobs Center for Theoretical and Computational Biology, Salk Institute for Biological Studies, La Jolla, CA 92037, USA; 2 Helen L Dorris Neuropsychiatric Disorder Institute and Department of Molecular Biology, The Scripps Research Institute, La Jolla, CA 92037, USA; 3 Department of Mathematical Sciences, Clemson University, Clemson, SC 29634, USA

## Abstract

**Background:**

Existing quantitative models of mouse cerebral cortical development are not fully constrained by experimental data.

**Results:**

Here, we use simple difference equations to model neural progenitor cell fate decisions, incorporating intermediate progenitor cells and initially low rates of neural progenitor cell death. Also, we conduct a sensitivity analysis to investigate possible uncertainty in the fraction of cells that divide, differentiate, and die at each cell cycle.

**Conclusion:**

We demonstrate that uniformly low-level neural progenitor cell death, as concluded in previous models, is incompatible with normal mouse cortical development. Levels of neural progenitor cell death up to and exceeding 50% are compatible with normal cortical development and may operate to prevent forebrain overgrowth as observed following cell death attenuation, as occurs in caspase 3-null mutant mice.

## Background

Essentially every excitatory neuron in the cerebral cortex is born from a heterogeneous pool of mitotic cells (referred to collectively as neural progenitor/precursor cells (NPCs)) in the embryonic ventricular zone (VZ) [1-4]. During a 'neurogenic interval' in mouse – commencing at embryonic day (E)10 in the rostro-medial cortex and concluding at E18 in the caudo-lateral cortex [5-7] – the founding NPC population expands through proliferative divisions until it is exhausted by terminal differentiation and programmed cell death (PCD). NPC proliferation must be balanced with the operation of PCD to produce a sufficient, but not supernumerary, neuronal population. Understanding of the cellular and genetic mechanisms controlling the size of the cerebral cortex, among the most notable distinctions of the brain's evolution [8,9], could benefit from an accurate quantitative model of the fate decisions made by NPCs.

Initial models of mouse NPC fate decisions are insufficiently constrained because it was assumed that cell divisions outside of the VZ do not contribute cortical neurons [10]; however, it is now clear that non-VZ mitoses contribute significant numbers of cortical neurons [11-13]. Earlier models also lacked direct measurement of an important parameter, the founding NPC population size, instead relying on two related but less direct measurements [14]: changes in the size of the VZ (from which the number of NPCs is extrapolated) and the fraction of cells emigrating from the VZ (from which VZ neuronal output is calculated). Direct measurement of the size of the founding NPC pool [15,16] provides a more accurate description than extrapolation and addresses a limitation of early models. These data are employed to constrain new models as pursued here.

A direct quantitative implication of non-VZ neuronal production is that additional PCD will be required to offset additional neuronal production. Reports of previous models contend that NPC death is an insignificant component of neurodevelopment [17-19] despite empirical data that are consistent with significant NPC death. Data supporting much higher levels of NPC death than proposed in prior models were first reported using a sensitive DNA end-labeling technique, '*in situ *end-labeling plus' (ISEL+), and ligation-mediated PCR [20-24]. Additional support came from recent analyses of NPC progeny, marked using a genomically encoded lineage tracer; here, the progeny clone size was found to diminish markedly at E14 [25]. Perhaps most compelling, deletion of numerous pro-cell death genes, including those encoding caspase 3 [26], caspase 9 [27], APAF1 [28], Bax, Bak [29], and Pten [30], as well as novel molecules like ephrins [31], all lead to brain overgrowth phenotypes. Conversely and consistently, null mutations in pro-survival genes (for example, those encoding Bcl-x [32], Survivin [33] and Mcl-1 [34]) lead to smaller brains. Given this large body of empirical evidence, new models should account for the more extensive operation of PCD during cortical neurogenesis.

Here we report new quantitative models that incorporate new data and are consistent with cortical PCD empirical evidence. In particular, with respect to total neuronal production, we demonstrate a clear requirement for substantial NPC death during mouse cerebral cortical development. These models further provide a quantitative explanation of neurodevelopmental cortical overgrowth phenotypes produced by PCD attenuation as observed in caspase 3-null mutant mice.

## Results

### Simple models of NPC fate decisions require intermediate levels of cell death

Using cumulative labeling of newly synthesized DNA and explicit counting of each labeled cell migrating from the VZ and subventricular zone, the fraction of terminally differentiating cells has been measured experimentally [14,18]. We denote these fractions *q*_*i*_, where *i *= 1, 2,..., 11 for the 11 cell cycles (CCs) estimated to occur during the neurogenic interval (Table 1). These data, *q*_*i*_, and the estimated initial population of NPCs (*P*_0_), allow us to determine, through mathematical modeling, what fractions of NPC death at each CC, *d*_*i *_for *I *= 1, 2,..., 11, yield VZ output consistent with experimental counts of the total number of excitatory neurons in the mature mouse cerebral cortex. To this end, we require an estimate for the plausible range of total VZ neuronal output.

**Table 1:** Model parameters and constraints

Parameter	Symbol	Values	References
Founding NPC population	*P*_0_	5 to 6 × 10^5^	[15,16]
NPC CC number	*i*	1 to 11	[6]
Fraction of NPCs that become newly	*q*_*i*_	*q*_1 _= 0.005; *q*_2 _= 0.04;	[14,37]
post-mitotic neurons		*q*_3 _= 0.09;*q*_4 _= 0.14;	
		*q*_5 _= 0.21;*q*_6 _= 0.31;	
		*q*_7 _= 0.42; *q*_8 _= 0.54;	
		*q*_9 _= 0.69;*q*_10 _= 0.84;	
		*q*_11 _= 1.0	
Fraction of NPCs that die at *i*^*th *^CC	*d*_*i*_	5% to >50%	[12,13,17]
After *i*^*th *^CC, number of new:			
Post-mitotic cells	Q_*I*_	Model specific,	
NPCs	P_*I*_	see text	
Dying daughter cells	D_*I*_		
Total neurons produced from NPCs		1 × 10^7 ^to 2.72 × 10^7^	See text
	VZ output		
Cortical neurons		10 to 16 × 10^6^	[53,54]
Cortical interneurons		15 to 30% of cortical neurons	[20,24,35]
Post-natal cell death		30 to 50% of cortical neurons	[20,24,55]
Fraction of IPCs at each CC	*q'*_*i*_	*q'*_1 _= 0; *q'*_2 _= 0; *q'*_3 _= 0;	[10,12,13]
		*q'*_4 _= 0; *q'*_5 _= 0.12;	
		*q'*_6 _= 0.12; *q'*_7 _= 0.16;	
		*q'*_8 _= 0.16; *q'*_9 _= 0.54;	
		*q'*_10 _= 0.54; *q'*_11 _= 0.54	

The total number of neurons in the adult mouse cerebral cortex has been estimated at between 1.0 × 10^7 ^and 1.6 × 10^7 ^(Table 1 and references therein), and we use this estimate to constrain simulations of VZ output. Approximately 15 to 30% of the estimated total neuronal population consists of inhibitory interneurons. In addition, the estimate of 1.0 to 1.6 × 10^7 ^neurons includes cell loss that is due to 30 to 50% post-mitotic (non-VZ) cell death (Table 1 and references therein). Taking these figures into consideration, we can determine a range for plausible VZ output as follows. First, a lower bound on VZ output corresponds to taking the low estimate of 1.0 × 10^7 ^neurons and assuming that 30% of these are interneurons and that there was only 30% post-mitotic death; that is, a lower bound on VZ output is ((1.0 × 10^7^) × (1 – 0.3))/(1 – 0.3) = 1.0 × 10^7^. Similarly, an upper bound on VZ output follows from taking 1.6 × 10^7 ^neurons and assuming that 15% are interneurons and that 50% post-mitotic cell death occurred; thus, a plausible upper bound on VZ output is ((1.6 × 10^7^) × (1 – 0.15))/(1 – 0.5)) = 2.72 × 10^7^.

Therefore, data suggest that an accurate quantitative model of mouse cerebral cortical neurogenesis should yield a plausible range of VZ output between 1.0 × 10^7 ^and 2.72 × 10^7^. However, the published estimate of 140 progeny per founding NPC [18] corresponds to 7.7 × 10^7 ^NPC progeny produced by 5.5 × 10^5 ^founding NPCs. NPC death was presumed negligible when calculating this progeny-per-NPC estimate and it cannot be reconciled with the maximum plausible VZ output.

**Figure 1:**
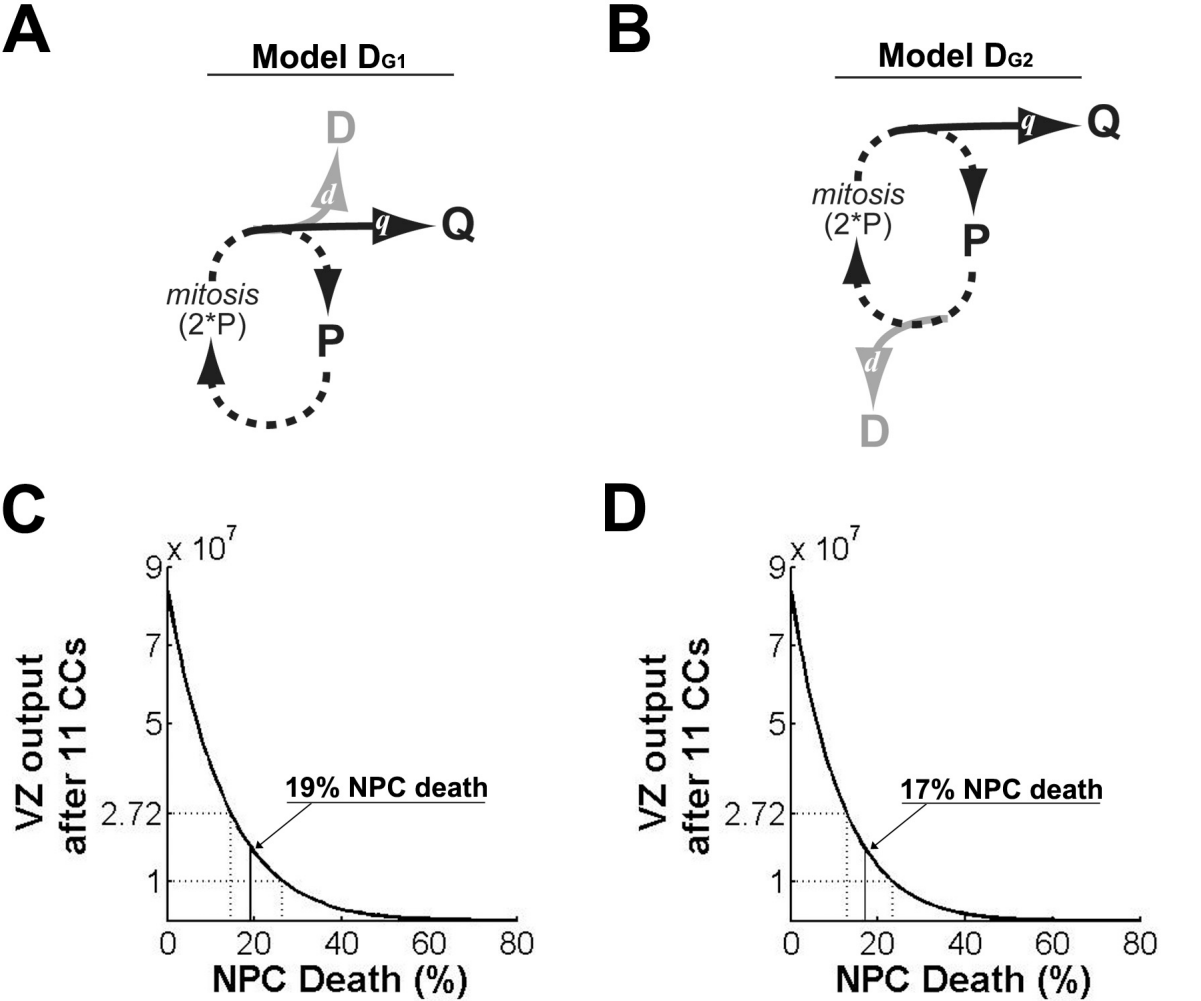
**Appropriate ventricular zone (VZ) output requires greater than 5% neural progenitor cell (NPC) death**. **(A, B) **Two simple cell decision paradigms are used to identify a plausibility window and calculate viable levels of NPC death. Beginning at 'P' and following the arrows clockwise, in model D_G1 _(A) the NPC population *P*_*i*-1 _is doubled at the *i*^*th *^mitosis, then NPC death, 'D,' and differentiation, 'Q,' are imposed, so that the P population for the subsequent cell cycle (CC) is given by *P*_*i *_= 2(1 - *d*_*i *_- *q*_*i*_)*P*_*i*-1_. (B) In model D_G2 _dying NPCs are removed from the NPC population prior to doubling the population, and *P*_*i *_= (1 - *q*_*i*_)2(1 - *d*_*i*_)*P*_*i*-1_. **(C, D) **Model output from *P*_0 _= 550,000. VZ output (total Q cells, y-axis) is plotted after 11 CCs when the indicated fraction of NPCs die at each CC (Death, x-axis). The window of plausible VZ output, between 1 × 10^7 ^and 2.72 × 10^7^, is indicated by dashed lines. (C) Using model D_G1_, the center of the plausible VZ output range (total Q cells = 1.86 × 10^7^) corresponds to 19% NPC death, as indicated by the solid line that descends from plotted VZ output to the x-axis. (D) Similarly, the center of the plausibility window using model D_G2 _corresponds to 17% NPC death.

To address this apparent contradiction, we calculated VZ output at various levels of NPC death using two related models of daughter cell fate decision-making (Figure 1A,B; Materials and methods). Model D_G1 _is derived from the experimental observation that NPC cell death occurs during the G1 phase of the CC [35], whereas model D_G2 _is more similar to existing models [17,18]. In both, the residual proliferative population before the *i*^*th*^CC, denoted *P*_*i*-1_, is doubled at mitosis. In model D_G1 _(Figure 1A), no cells die prior to cell division while in model D_G2_, death is imposed before each division. As a simple consequence, when *d*_*i *_is uniformly constant, model D_G1 _with founding population *P*_0 _is equivalent to model D_G1 _with founding population *P*_0_*/*(1-d_*i*_). Thus, without NPC death (*di *= 0 for all *i*), the same VZ output is calculated for each model: 8.4 × 10^7^, or 153 progeny per NPC. Assuming *d*_*i *_= 0.05 for all *i*, as measured using the less-sensitive technique terminal dUTP nick-end labeling (TUNEL) [35], VZ output for D_G1 _and D_G2 _are 5.8 × 10^7 ^and 5.5 × 10^7^respectively, both more than twice the plausible upper bound on VZ output (2.72 × 10^7^) established above.

Caviness and colleagues [18] calculated that a founding population of 2.5 × 10^5 ^NPCs was compatible with their model. The plausibility window when *P*_0 _= 2.5 × 10^5 ^accommodates between 5 and 17% NPC death for model D_G1 _and between 4 and 15% NPC death for model D_G2 _(data not shown). These calculations suggest that a significant reduction of the founding NPC pool could be consistent with lower levels of cell death during development; however, this explanation is not consistent with two independent measurements of the founding NPC pool size [15,16].

Our models allow us to view the range of plausible VZ output as a function of PCD over the course of 11 CCs, and we refer to this correspondence as the 'plausibility window.' For example, as shown in Figure 1C,D, a plausible range of VZ output is observed when 13% <*d*_*i *_< 26% for all *i*. Because of the delayed depletion of the proliferative population, the corresponding range of plausible *d*_*i *_values in model D_G1 _is broader than that calculated using model D_G2 _(that is, 15 to 26% versus 13 to 23%, respectively). Neither model D_G1 _or D_G2 _matches the experimental measurements of either 5% NPC death using TUNEL or 50% NPC death using ISEL+; however, these calculations do demonstrate that 5% NPC death is too low to calculate VZ output adequately in the normal mouse brain.

### Sensitivity analysis of model DG_1_

Measurements of CC duration together with the fraction of NPCs that differentiate at experimentally defined ages permits extrapolation of model parameter *q*_*i *_for each *i *[14]. Sensitivity analysis [36] provides a means of determining the relationship between inherent uncertainty in these estimates of *q*_*i *_and uncertainty in simulated VZ output.

**Figure 2:**
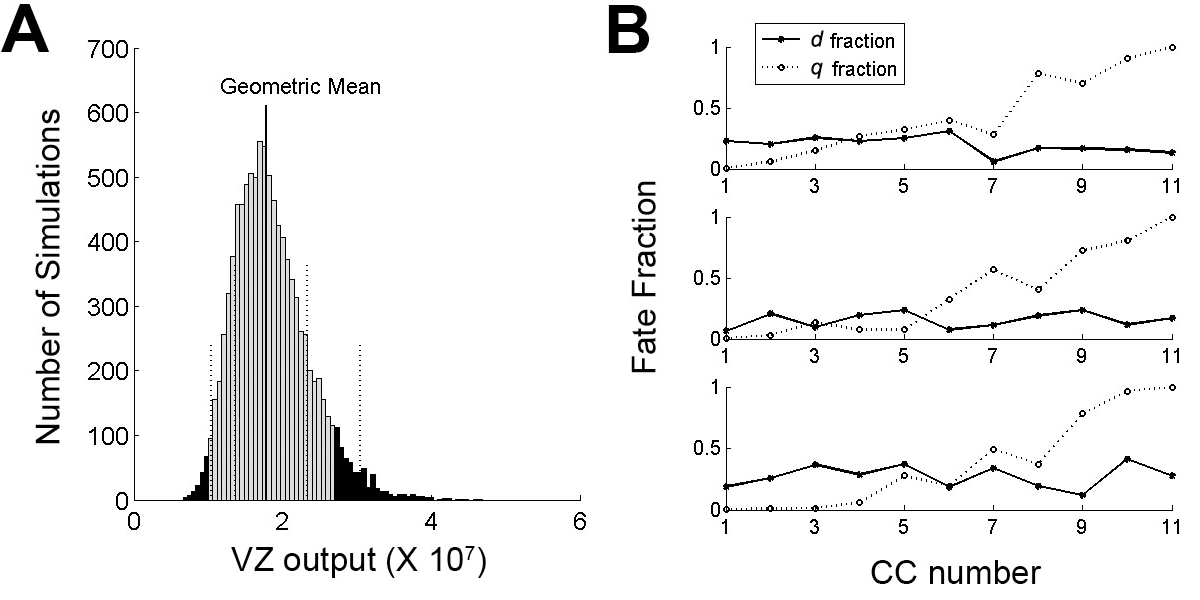
**Model D_G1 _is insensitive to perturbations in *q*_*i *_and *d*_*i*_**. **(A) **Most (>90%) model realizations give plausible ventricular zone (VZ) output (gray bars). The distribution of 10,000 simulations of VZ output (where  = 0.19 for all *i*) is plotted as a histogram. Simulation outputs follow a log-normal distribution. The solid vertical line indicates the geometric population mean, and dashed vertical lines indicate 1 and 2 standard deviations from the mean. **(B) **Three examples of points in 22-dimensional parameter space where plausible VZ output is observed. The *q *fractions are plotted along a dashed line and *d *fractions are plotted along a solid line. The upper panel is from a bin below the mean, the center panel is from the bin at the mean, and the lower panel is from a bin above the mean. CC, cell cycle.

Using a Monte Carlo approach, we sampled each *q*_*i *_and *d*_*i *_as normally distributed random variables with means  and  taken from [14,37] and variance given by  and  (Materials and methods). For example, for  and *ν*_1 _= 0.5 we have *q*_*i *_~*N*(0.42, 0.122). Sampling the 22-dimensional parameter space for *q*_*i *_and *d*_*i*_, and then computing VZ output for each sample point in parameter space, allowed us to determine that *ν*_1 _= *ν*_2 _= 0.5 gives plausible VZ output for >90% of the model realizations (Figure 2A).

For *ν*_1 _= *ν*_2 _= 0.5, we computed the first-order sensitivities of VZ output to variation in each *q*_*i *_and *d*_*i *_(Materials and methods). The first-order sensitivities  and  are measures of how uncertainty in each *q*_*i *_and *d*_*i *_drives uncertainty in VZ output. For example, when  is close to zero for a given *q*_*i*_, significant variation in VZ output can still occur despite fixing this *q*_*i*_. We find that death rates in the first few CCs and differentiation rates in the middle CCs can account for the most significant portion of model variability; yet even the largest  and  are only approximately 10% (Table 2). As such, variation in VZ output is hardly attributable to variation in any one *q*_*i *_or *d*_*i*_. An obvious explanation is that, given exponential growth conditions, outlying values of either *q*_*i *_or *d*_*i *_in early CCs are easily compensated by outliers in later CCs. Interestingly, significant variation in *q*_*i *_and *d*_*i *_from the mean values  and  can still yield plausible VZ output (Figure 2B).

**Table 2:** Representative sensitivity analysis

	Cell cycle										
	1	2	3	4	5	6	7	8	9	10	11

S*q*_*i*_	0.0007	0.0041	0.0161	0.03	0.055	0.0974	0.065	0.0314	0.0079	0.0012	0.0005
S*d*_*i*_	0.109	0.1105	0.1144	0.0914	0.0728	0.0629	0.0452	0.0151	0.0038	0.0006	0.0004

### Intermediate progenitor cells alter the plausibility window

NPC mitoses occur on the ventricular surface, but 'non-surface' mitoses are also observed in the developing cortex. Initially, these cell divisions were erroneously considered non-neuronogenic [10], but proliferative intermediate progenitor cells (IPCs) – daughter cells of NPCs that have migrated to the subventricular zone and intermediate zones, and are immunoreactive for the transcription factor Tbr2 – are now known to contribute additional excitatory neurons to the mouse cerebral cortex [11-13].

**Figure 3:**
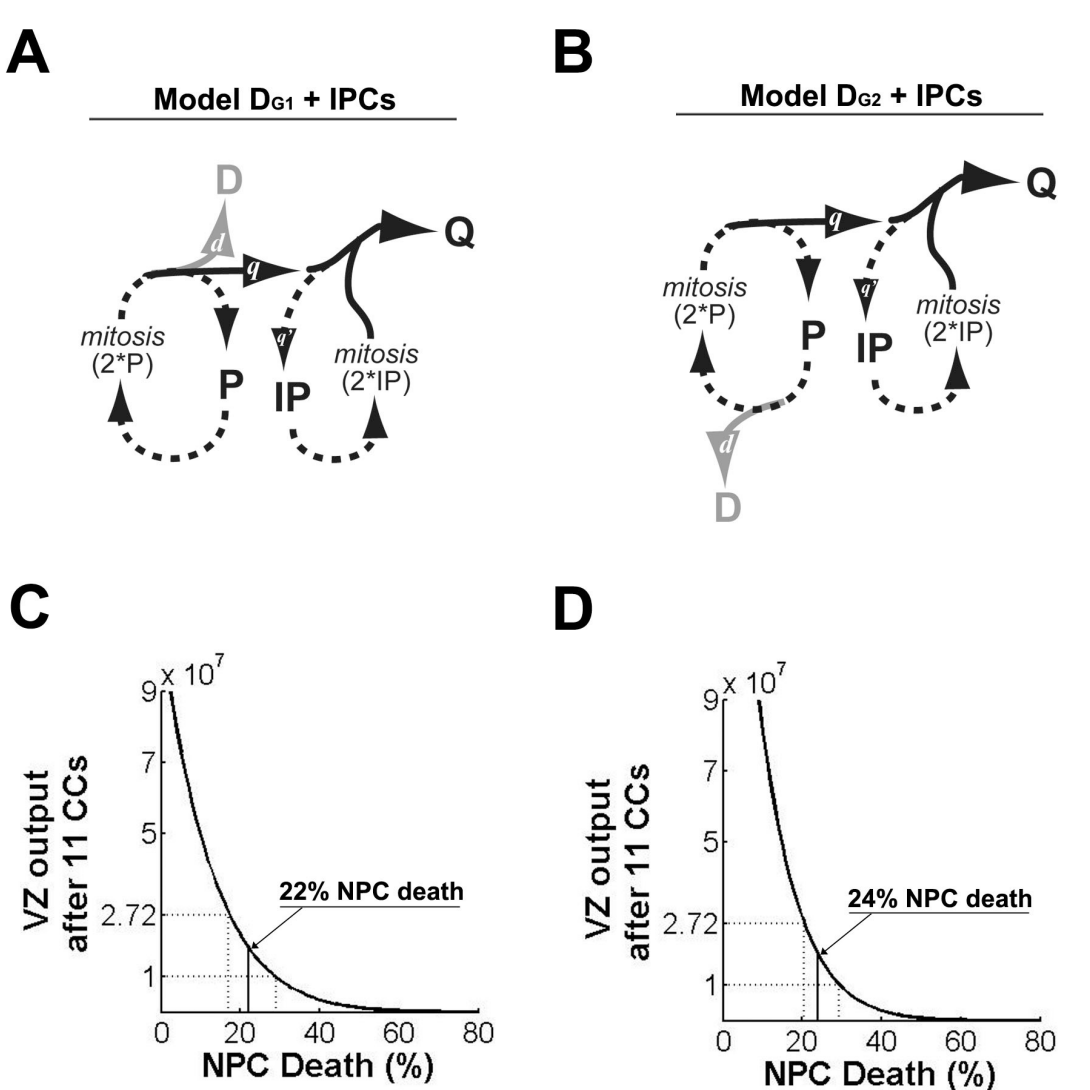
**Intermediate progenitor cells (IPCs) modestly increase the requirement for neural progenitor cell (NPC) death**. **(A, B) **IPCs (IP) are incorporated into (A) model D_G1 _and (B) model D_G2 _similarly. A fraction of Q cells (*q*^'^) become IPCs, undergo one CC (2 *IP), then re-enter the Q population. **(C, D) **Ventricular zone (VZ) output plotted as described in Figure 1. (C) In model D_G1 _the plausibility window is centered at 22% NPC death. (D) In model D_G2 _the plausibility window is centered at 24% NPC death.

To determine how IPCs may affect the level of NPC death required for plausible VZ output, IPC subpopulation levels were extrapolated from the literature (Table 1 and references therein) and these constraints were incorporated into both models. We take a fraction of the Q cell population after the *i*^*th *^division – that is, we denote *q*_*i*_'*Q*_*i *_as the size of an IPC subpopulation – and these cells undergo an additional CC, doubling their contribution to the total Q population (Figure 3A,B). In initial modeling, we assume that additional IPC CCs and IPC death either do not occur or at least offset each other, and we include those IPCs generated after the last NPC CC in the VZ output calculated for CC_11_. With the inclusion of IPCs, VZ output without NPC death increases to 1.1 × 10^9 ^in model D_G1 _and more than doubles to 2.1 × 10^9 ^in model D_G2 _(Figure 3C,D). With 5% NPC death, model D_G1 _calculates VZ output to be 7.3 × 10^8 ^and model D_G2 _calculates 1.3 × 10^9 ^(Figure 3C,D). Since the *q*_*i*_' fraction is largest in the last three CCs, IPCs have a relatively small effect on the plausibility window; it expands slightly to accommodate 17 to 29% NPC death in model D_G1 _and 20 to 29% NPC death in model D_G2_. Importantly, a larger IPC population (from a larger IPC fraction or additional IPC CCs without IPC death) could increase, but not decrease, the requirement for NPC death.

Cortical development with 5% NPC death at early CCs requires more than 50% NPC death at later CCs

A primary objection to the idea of substantial NPC death is the intuition that ≥ 50% NPC death throughout neurodevelopment would preclude expansion of the NPC pool. However, the level of NPC death need not be constant at each CC. For example, ISEL+ labels approximately 5% of cells at E10 and the percentage increases significantly thereafter [20,35]. Moreover, NPC lineage analyses using a genomically encoded marker found that clone size increased during early development but then diminished after E14 [25], suggesting significant cell death with corpse elimination at later, but not earlier, CCs. These empirical observations are also consistent with increased model sensitivity to the levels of death at earlier CCs (Table 2). Together, these data suggest that early 'expansion' CCs occur; initially, low PCD levels – for example, setting *d*_*i *_= 0.05 for 1 ≤ *i *≤ 4 provides for four expansion CCs – further constrain VZ output. Here we calculate the corresponding amount of NPC death required during later CCs for plausible VZ output.

**Figure 4:**
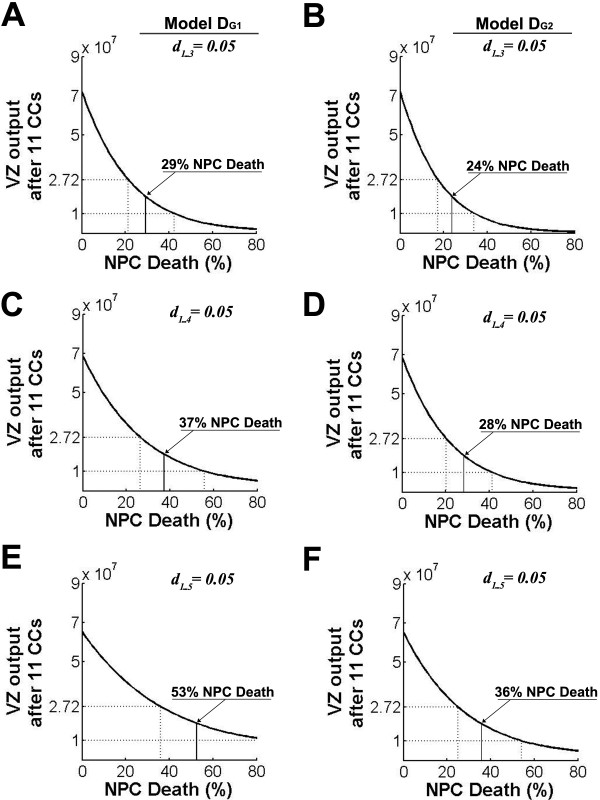
**Expansion cell cycles permit much more neural progenitor cell (NPC) death at later cell cycles (CCs)**. Ventricular zone (VZ) output plotted as described in Figure 1. During expansion CCs, *d*_*i *_= 0.05; NPC death during subsequent CCs is indicated on the x-axis. **(A, B) **After three expansion CCs the plausibility window is centered at 29% using model D_G1 _(A) and 24% using model D_G2 _(B). **(C, D) **After four expansion CCs the plausibility window is centered at 37% using model D_G1 _(C) and 28% using model D_G2 _(D). **(E, F) **After five expansion CCs the plausibility window is centered at 53% using model D_G1 _(E) and 36% using model D_G2 _(F).

The fourth neurogenic CC takes place on E12, a time when significant ISEL+ labeling was observed [20]. VZ output with three, four, or five expansion CCs amplifies differences between models D_G1 _and D_G2_. In model D_G1 _with three expansion CCs, the plausibility window permits 21 to 42% NPC death; four expansion CCs permit 26 to 56% NPC death; and five expansion CCs permit 36 to >80% NPC death (Figure 4A,C,E). Using model D_G2_, the plausibility window is less broad and accommodates less NPC death. After three expansion CCs the D_G2 _plausibility window permits 17 to 34% NPC death; after four expansion CCs it permits 20 to 41% NPC death; and after five expansion CCs it permits 25 to 54% NPC death (Figure 4B,D,F).

We note here that inclusion of four or five expansion CCs makes model D_G1 _and model D_G2 _readily distinguishable from one another. This distinction is a consequence of the differential involvement of cell death when expansion CCs begin and end, and illustrates some additional sensitivity to the size of the founding NPC population. As described above, during the first CC in model D_G2 _the founding population is reduced before mitosis; therefore, for uniformly constant cell death, model D_G2 _with initial population *P*_0 _is equivalent to model D_G1 _with initial population *d*_1_*P*_0_. Although this distinction leads to only subtle differences in most simulations, when considering the relationship between expansion CCs and plausible levels of NPC death, model D_G1 _with four or five expansion CCs illustrates additional potential for biological variation.

Early expansion CCs and IPCs together are most compatible with experimental data and further support high levels of NPC death

**Figure 5:**
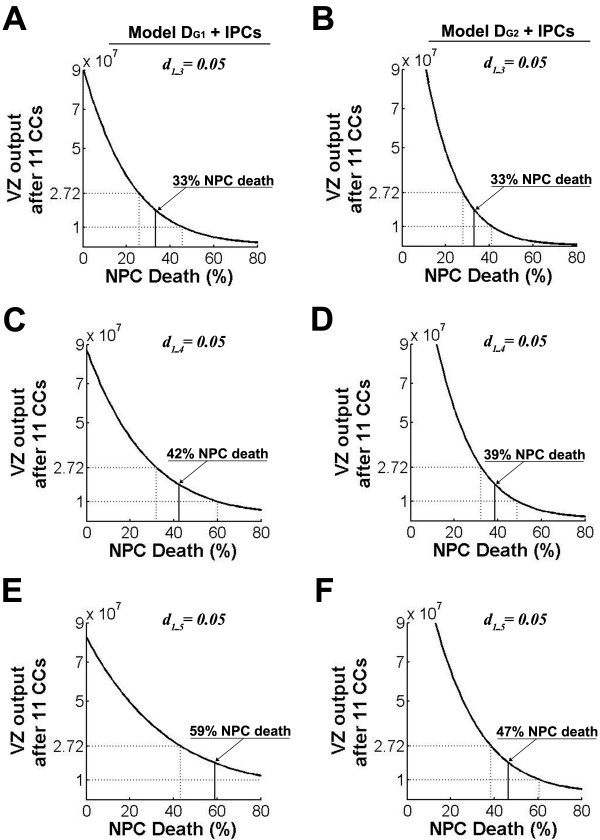
**Constrained models require substantial levels of progenitor cell (NPC) death**. Ventricular zone (VZ) output as plotted in Figure 1, including intermediate progenitor cells (IPCs) as in model D_G1 _(Figure 3A) and model D_G2 _(Figure 3B). **(A, B) **After three expansion cell cycles (CCs) the plausibility window is centered at 33% using model D_G1 _(A) and 33% using model D_G2 _(B). **(C, D) **After four expansion CCs the plausibility window is centered at 42% using model D_G1 _(C) and 39% using model D_G2 _(D). **(E, F) **After five expansion CCs the plausibility window is centered at 59% using model D_G1 _(E) and 47% using model D_G2 _(F).

Both expansion CCs and IPC CCs occur during normal mouse brain development. When NPC population kinetics include expansion CCs (as shown in Figure 4) and IPCs (as shown in Figure 3), the plausibility window is broadest. For model D_G1 _with IPC CCs, three expansion CCs permit 26 to 46% NPC death and four permit 32 to 60% NPC death (Figure 5A,C). After five expansion CCs, VZ output already exceeds the minimum calculated estimate of cortical neurons (data not shown), with = 43% NPC death required during the remaining CCs to restrain VZ output (Figure 5E). As above (Figure 3), model D_G2 _with IPC CCs yields a lower and narrower range of NPC death levels: 28 to 41% with three expansion CCs, 32 to 49% with four expansion CCs, and 38 to 61% with five expansion CCs (Figure 5B,D,F).

## Discussion

Cell death amongst NPCs is a prominent feature of neurogenesis in other regions of the nervous system (for example, retina; reviewed in [38,39]); however, the amount of NPC death during mouse cerebral cortical development is debated [40,41]. Employing published cell counts to estimate ranges of neuronal population size in the mouse cerebral cortex, we calculated a plausible range of VZ output for normal mouse neurodevelopment. Models D_G1 _and D_G2 _use simple difference equations to capture essential features of previous probabilistic models. Model D_G1 _with three to five early expansion CCs, a pool of IPCs, and NPC death near 50% incorporates the most experimentally observed constraints and is consistent with NPC death levels as observed in ISEL+ analyses [22,42,43].

### Comparison with contemporary models

Apart from initial modeling by Takahashi, Caviness, and colleagues (referenced throughout), Gohlke, Faustman, and colleagues [17,44] have used a Kolmogorov forward equation to compute the probability distribution for mouse VZ output explicitly, as a continuous-time Markov chain. In contrast, we estimate this same probability distribution by repeatedly simulating a deterministic difference equation with random perturbations taken from assumed distributions. This allows us to illustrate how significant variation in *q*_*i *_and *d*_*i *_fractions, over the course of 11 CCs, can occur and still yield plausible VZ output (Figure 2B). In population measurements using stereological counting, variation at the level of subpopulations of NPCs would probably go unnoticed, yet this may be an important feature of cerebral cortical development. While such variation is implicit in the Kolmogorov forward equation approach, Monte Carlo allows us to view sample trajectories that illustrate this variation.

The Gohlke models also observe that the original Takahashi models lead to VZ output that exceeds experimental counts of cortical neurons by at least threefold [44]. However, these authors use two additional parameters (in addition to low-level NPC death) to reduce cortical neuron production to plausible levels: a diminished growth fraction insofar as not all VZ cells are progenitor cells and a clearance time for dying NPCs. Many NPCs co-label for bromodeoxyuridine and ISEL+ [20,24,42]; therefore, Gohlke and colleagues have likely recast some additional NPC death as a 'diminished' growth fraction. These parameters have the effect of reducing the proliferative population from which any given *q *fraction is taken. Consequently, the overproduction observed using the Takahashi model is limited and plausible levels of VZ output are obtained from the Gohlke mouse model [17,44].

The Gohlke mouse model is reportedly compatible with levels of NPC death up to 24% [17]. This value is in good agreement with 13 to 26% NPC death calculated using models D_G1 _and D_G2 _(Figure 1). Despite this, Gohlke and colleagues [17,45] report subsequent model analysis using NPC death rates at or near 0% NPC death. The contrasting higher levels of NPC death required in Gohlke models of primate cortical neurogenesis [45], relative to murine cortical neurogenesis, may simply reflect an underestimation of mouse NPC death.

### TUNEL underestimates NPC death

A strong implication of all modeling experiments presented here is that TUNEL significantly underestimates NPC death during mouse cerebral cortical development. This reflects technical differences in sensitivity between the two procedures, with ISEL+ being approximately ten times more sensitive than the originally reported TUNEL technique [21,22]. ISEL+ detects more dying cells not only amongst NPCs, but also in other tissues like the thymus and small intestinal villus [43]. Consistent with a tenfold reduced sensitivity relative to ISEL+, TUNEL detects as few as one-tenth of the dying NPCs (5% versus 50%).

### Consideration of the caspase 3-deficient phenotype

In order to reconcile the approximately 5% NPC death proposed by prior models [18] with the forebrain overgrowth phenotype observed in caspase 3-deficient mice, it has been proposed that NPC death may occur normally in a small population of neuroepithelial stem cells or radial glia at an early age (<E12) [46]. The claim is that additional survival of a few such cells could underlie forebrain overgrowth in caspase 3-deficient mice because each of these individual cells might ultimately give rise to many neurons (approximately 140 according to estimates from concurrent models). However, this notion is inconsistent with experimental data demonstrating marked overgrowth of NPCs in caspase 3-null embryos by E12, before large numbers of NPC progeny emigrate to the cortex [26,42].

Our models are consistent with changes in NPC and neuronal populations that have been observed following experimental attenuation of PCD. For example, a 20% reduction in NPC death from the midpoint of any plausibility window corresponds to VZ output that exceeds the upper bound for that plausibility window. This is strikingly consistent with a 30% reduction in ISEL+ labeling of NPCs observed in caspase 3-deficient mice [42], where exceptional forebrain overgrowth is observed. We suggest that an *in vivo *correlate of 'exceeding the plausibility window' is forebrain overgrowth.

### Predictions derived from observing the plausibility window

The breadth of the plausibility window provides a comparative measure of model robustness with respect to viable levels of NPC death. In some scenarios the slope of VZ output is steep (Figure 1D), and a 10% difference in NPC death leads to marked differences in VZ output, while a similar 10% change has little impact on VZ output in models where the slope is less steep (for example, Figure 4E). Notably, early expansion CCs (Figure 4), rather than the size of the founding NPC population or additional IPC progeny (compare Figures 2 and 3), significantly extend the range of NPC death that is compatible with plausible VZ output. Although modeled during the first three to five CCs here because of experimental evidence for pre-E12 expansion CCs [20,25], transient low-level NPC death could theoretically operate during any CC. It is tempting to speculate that high levels of NPC death at later CCs follow from low levels of NPC death during early expansion CCs. A similar process has been reported in embryonic stem cells earlier in development [47,48] and may be related to a permissive decatenation checkpoint observed in NPCs [48].

One might predict that other perturbations leading to transient high levels of NPC death at early CCs could lead to low level NPC death at later CCs. In this scenario, developmental accommodation of atypical NPC death might occur at the level of stem cell niches [49,50], providing sufficient, but not necessarily 'normal,' VZ output. Perhaps local control of NPC death could insulate cerebral cortical development against genetic differences and chemical or environmental insults. Given genetic diversity among NPCs, produced in part by chromosomal aneuploidy [51] and retrotransposition [52], differences in NPC death amongst individuals suggests selection and/or survival mechanisms that influence the mosaic composition of an individual's cerebral cortex.

## Conclusion

Models D_G1 _and D_G2 _resolve discrepancies existing between previous models and experimental data; furthermore, these models provide a quantitative account for qualitative differences observed during PCD-attenuated cerebral cortical development. This theoretical framework should motivate additional experimental investigation of expansion CCs and reinterpretation of other cortical development phenotypes that measured PCD among NPCs using only TUNEL staining.

## Materials and methods

The order in which NPC death, differentiation, and proliferation are imposed alters VZ output, so it is natural to consider two related models. For the first model, D_G1_, the number of generated neurons at the *i*^*th *^CC is *Q*_*i *_= 2*q*_*i*_*P*_*i*-1_, where *P*_*i*-1 _is given by *P*_*i *_= (1 - *d*_*i*_)(1 - *q*_*i*_)2*P*_*i*-1_. Here, (1 - *q*_*i*_)2*P*_*i*-1 _is the number of non-emigrating daughter cells after the *i*^*th *^division and *d*_*i*_(1 - *q*_*i*_)2*P*_*i*-1 _of these die, leaving *P*_*i*_(1 - *d*_*i*_)(1 - *q*_*i*_)2*P*_*i*-1 _to divide again. Alternatively, imposing death before imposing differentiation gives *Q*_*i *_= *q*_*i*_(1 - *d*_*i*_) 2*P*_*i*-1 _while, again, *P*_*i*_(1 - *d*_*i*_)(1 - *q*_*i*_)2*P*_*i*-1 _many NPCs go through the *i*^*th *^CC. We refer to this model as D_G2_. In each of these two related models, the total VZ output over 11 NPC CCs is . Viewing *VZ *= *VZ*(***q***, ***d***) as a function of all *q*_*i *_and *d*_*i *_– that is, ***q ***= (*q*_*i*_) and ***d ***= (*d*_*i*_) are 11-dimensional vectors – allows us to explore how total VZ output is sensitive to variation in *q*_*i *_and *d*_*i*_.

Defining  and  as the mean differentiation and death rates, we generate random values ***q ***= (*q*_*i*_) and ***d ***= (*d*_*i*_) using a normal distribution. As variances we use  and  so that when either *q*_*i *_or *d*_*i *_are close to 0 or 1, sampling rarely provides values that are nonbiological; that is, less than 0 or greater than 1. To indicate this distribution, we write  and . Then, following Saltelli *et al*. [36], we define first-order sensitivities:



and



where *Var *[*VZ*(***q***, ***d***)] is the variance in VZ output that arises when all *q*_*i *_and *d*_*i *_are randomized (for example, 10,000 runs) and where *Var *[*E*[*VZ*(***q***, ***d***|*q*_*k*_)]], for example, is the variance in expected VZ output that arises when, for some fixed *k*, all *q*_*i *_with *i *≠ *k *are allowed to vary. That is, to compute *Var *[*E*[*VZ*(***q***, ***d***|*q*_*k*_)]], *q*_*k *_is fixed repeatedly while  for all *i *≠ *k *and  for all *i *= 1, 2,..., 11. Then, the average (expected) VZ output is taken, and this entire process is repeated (for example, approximately 100 times) to determine the variance of such an average.

Calculations were performed using MatLab version 7.7.0 (Mathworks, Natick, MA, USA) as detailed in the text. Figures were prepared using Illustrator and Photoshop (Adobe Systems Inc., San Jose, CA, USA). The MatLab scripts are available as additional files 1 and 2.

## Abbreviations

CC: cell cycle; E: embryonic day; IPC: intermediate progenitor cell; ISEL+: *in situ *end-labeling plus; NPC: neural progenitor/precursor cell; PCD: programmed cell death; TUNEL: terminal dUTP nick-end labeling; VZ: ventricular zone.

## Supplementary Material

Additional file 1 **matlab script.** Click here for file

Additional file 2 **matlab script.** Click here for file
